# Editorial: Harnessing placebo mechanisms

**DOI:** 10.3389/fpsyt.2022.1022762

**Published:** 2022-09-12

**Authors:** Chamindi Seneviratne, Jason Noel, Patricia D. Franklin, Luana Colloca

**Affiliations:** ^1^Department of Psychiatry, University of Maryland School of Medicine, Baltimore, MD, United States; ^2^The Institute for Genome Sciences, University of Maryland School of Medicine, Baltimore, MD, United States; ^3^Department of Pharmacy Practice and Science, University of Maryland School of Pharmacy, Baltimore, MD, United States; ^4^Department of Partnerships, Professional Education and Practice, University of Maryland School of Nursing, Baltimore, MD, United States; ^5^Department of Pain and Translational Symptom Science, University of Maryland School of Nursing, Baltimore, MD, United States; ^6^The Placebo Beyond Opinion Center, University of Maryland School of Nursing, Baltimore, MD, United States

**Keywords:** placebo, nocebo, pain, genetics, SIPS

## Introduction

The placebo phenomenon is receiving increasing attention because of the high translational value of basic research that can effectively translates into better study designs and symptoms management (1). This Theme Issue collection represents current trends in placebo research by focusing on two main strategies: (1) characterizing temporal effects, and (2) identify neuropsychobiological factors that can be used to subgroup individuals in clinical research for personalized treatments or interventions. The present collection predominantly focuses on placebo and nocebo effects associated with pain-related outcomes that were presented at the 3rd International conference of the Society for Placebo Studies (SIPS) in 2021. The first major section comprises of six studies that examined placebo and nocebo effects, with a focus on contextual features and individual predictors to be considered in designing rigorous research in these areas. The second major section is comprised of another six studies that investigated the potential for use of techniques that elicit nocebo and or placebo responses in clinical practice, with a focus on treating acute and chronic pain.

The Special Topic issue begins with an article by Cornell et al. reporting results of the SIPS 2021 Conference. In keeping with an important objective of this meeting, senior faculty worked with graduate and undergraduate students to design a post conference evaluation. These students had provided essential support in operationalizing and facilitating the translation of a traditional design to a virtual conference platform and took the lead in analyzing data collected by the hosting platform throughout the conference. As the authors discuss, having quantitative data that measured individual attendee activity during and across the three-day conference proved valuable in describing the level and degree of participation. Descriptive analyses of quantitative data collected during the conference indicated a highly successful program as well as revealed and implications for future, scientific meetings. Specifically, the results identified challenges of creating and sustaining meaningful networking in a virtual platform within the context of an international meeting where attendees represented multiple time zones. The authors also identified and discussed issues that influenced the design and evolution of this meeting, particularly the COVID-19 pandemic.

## Considerations for designing rigorous research on placebo and nocebo effects

### The role of contextual factors on placebo and nocebo effects

Contextual factors (CF) are various elements deriving from a patients' interactions with practitioners and the therapeutic arena that influence disease processes and therapeutic outcomes (2, 3). Whilst positive CF may induce placebo effects, negative CF may induce nocebo effects resulting in adverse effects (4, 5). However, studies assessing specific CF have yielded mixed results on their contribution to placebo/nocebo effects (2, 6, 7). This heterogeneity in outcomes have prompted investigating the context of how the CFs are assessed. To elucidate these mechanisms further, the first three manuscripts consider whether and how temporal expectations modulate placebo hypoalgesia and nocebo hyperalgesia. A study by Rosenkjær et al. from Aarhus University in Denmark and the Harvard Medical School in the US, examines the temporal relationship of expectations to placebo effects. Specifically, whether and how the temporal development of expectations affects research subjects' experiences over time. The results of the qualitative and quantitative data presented in this manuscript indicate that the timing of the assessment of expectations in placebo trials is a crucial feature of studying and clarifying placebo effects. Next, a collaboration between Camerone et al. from Italy, Belgium and the United Kingdom, elaborates on the construct of temporal modulation in placebo and nocebo studies. The authors examined the modulation of nocebo effect, the onset of action, and time-course of nocebo hyperalgesia in a model of sustained pain. The results of this study inform the design of clinical trials that will expand understanding of treatment negative expectations and drug side effects. A third study (Benson et al.), conducted by a team of scientists from Essen University Hospital and Ruhr University in Germany, investigated effects of pre-treatment expectations on post-treatment perceived treatment efficacy. Benson et al. used an experimental model of visceral pain and measured the effects of pre-treatment expectation on post-treatment perceived treatment efficacy. Results confirmed individual's positive expectations and perceived symptom improvement facilitates treatment satisfaction. These three studies also have implications for improving treatment outcomes if clinicians have the knowledge and understanding of the relationship between treatment expectations on both placebo and nocebo effects.

Reinforcing expectancies have been shown to augment hypoalgesia in many previous work (8–10). Building upon these studies, Proulx-Bégin et al. from Université de Montréal and McGill University in Canada consider a proof-of-concept conditioning procedure based on a surreptitious augmenting intervention expectation as a method for enhancing hypoalgesic effect. While the study was conducted in a population of healthy volunteers, it provides a model for the investigation of conditioning to raise expectations in patients with chronic pain, and perhaps other chronic conditions.

### Individual predictors of placebo and nocebo effects

The placebo and nocebo effects are neuropsychobiological responses that are highly heterogeneous amongst individuals (11, 12). Recently, much attention has been directed toward identifying individual characteristics to broaden our understanding of individual differences in placebo/nocebo responses particularly in clinical settings. Two articles here contribute to the *personalized* approach to harnessing placebo/nocebo effects. Weng et al. from the Netherlands explore individual psychological predictors of generalization of nocebo and placebo effects within and across pain and itch modalities. Next, in a collaborative exploratory genome-wide association study (GWAS) by researchers from Germany, the UK, and the US, Vollert et al. revealed that the pain severity and pain frequency subscales are associated with distinct genetic loci, highlighting the need for replication studies to characterize neurobiological underpinnings.

## The use of placebo and nocebo effects in clinical practice

Increasingly, studies demonstrate the clinical effectiveness of placebo and nocebo responses. It is fitting, therefore, to understand the health care professionals' knowledge, perspectives and use of placebo and nocebo effects. The first study of this second section by Smits et al. reports findings from a cross-sectional survey of general practitioners in The Netherlands. The study revealed limited knowledge on use of placebo in practice as well as a pervasive perspective that use of placebo is necessarily “deceptive” and thus potentially unethical. This gap in understanding of placebo effect and placebo response impedes its application in clinical practice. For example, an important dimension to treating acute pain is appreciating the influence of preoperative mood and treatment expectations on postoperative pain. Stuhlreyer and Klinger, from Germany, found a strong relationship between these two variables and suggest a preoperative expectation management program focusing on the patient's emotional state has potential for significantly reducing post-operative pain. The study by Olliges et al. in Germany and Switzerland investigated the effect of open-label placebo in treating elderly knee pain associated with osteoarthritis. This study adds to the growing understanding that deception is not necessary to evoke placebo effects. Bedford et al. (United States), in their study on patients and clinicians' perspectives toward a pre-authorized concealed opioid taper. Chronic pain, such as osteoarthritis, also requires a complement of treatments. Prescribing therapeutic pain treatments without placing patients at risk of opioid addiction is an ongoing dilemma. Colloca et al. in the United States demonstrated how expectancies can be shaped to optimize patients' attitudes toward their need for opioid analgesics through educational interventions in participants who experienced trauma induced pain. The last article by Trakimas et al. in the United States, reports on their study to develop guidelines for opioid requirement following hospital discharge of patients who underwent surgery for head and neck cancer. Current post surgical opioid prescribing patterns are not having the desired effect in reducing risk for opioid dependence post-surgery; the authors highlight the need for guidelines for post-surgical opioid requirements and the potential use of conditioning therapy and placebo to augment limited use of opioids post-discharge.

## A source for placebo literature

An expertly curated bibliography is a valuable resource for scientists as well as practitioners. While the increasing collaborative and multidisciplinary research conducted in this field bodes well for expanding the science and ultimate translation to treatment, it also poses a challenge to conducting a search of the literature as a result of the multiple areas of science involved. This Special Topic Issue provides a bibliometric exploration of the placebo literature. The bibliometric analyses of the JIPS data base indicates positive growth in research programs, especially interconnections between research groups, areas for future developments, and implications for conducting a search of the literature.

## Final remarks

In conclusion, this collection of multifaceted studies presents valuable insights into ways in which scientific rigor in harnessing placebo effects can be strengthened in order to improve patient's outcomes. We would like to emphasize that this Theme Issue is a product of the 3rd SIPS Conference held virtually in May 2021 at University of Maryland, Baltimore, USA. It was an international scientific meeting designed to advance the science of placebo and nocebo research and apply this knowledge to treatment of alcohol and other substance use disorders as well as improve treatment of acute and chronic pain. It is well-established that the placebo/nocebo effects are complex, and the heterogeneity of the responses impedes our understanding of these effects. Recently, specific emphasis has been given toward addressing when or how should the placebo/nocebo effects studied to optimally capture the responses. This shift in paradigm has led to the immergence of numerous investigational strategies to harness placebo/nocebo effects overcoming heterogeneity. Conference presentations elucidated both the complexity of designing robust research programs on nocebo and placebo responses and effects, as well as its translation and application to clinical practices for improved risk reduction, treatment and management of pain and substance use disorders. The SIPS 2021 Conference full proceedings, including abstracts from junior scientists, may be found here (https://www.frontiersin.org/books/3rd_International_Conference_of_the_Society_for_Interdisciplinary_Placebo_Studies_SIPS_Harnessing/5009).

We invited senior scientists, who participated in workshop presentations at SIPS 2021 Conference, to submit manuscripts for this Special Topics Issue: (Harnessing Placebo Mechanisms). Scientists from European and North American countries responded. In some cases, the submitted work was a result of research collaborations and partnerships across Europe and between the United States and European countries, reflecting the growing collaboration in this field. Proposed articles had to be based on original research the author presented at the conference. Manuscripts were peer reviewed and selected to participate. While SIPS 2021 conference featured a few studies on SUD and a plenary presentation on alcohol use disorder (AUD), placebo studies in SUD/AUD are underrepresented in this special issue as in elsewhere. We received overwhelming positive feedback from those who attended the conference. A few examples of feedback are shared here in the article by Cornell et al. With this in mind, we hope that the present collection of studies will reignite enthusiasm for placebo research amongst the scientific community. Finally, we would like to thank all attendants, junior and senior speakers for their valuable contribution to the SIPS Conference, reviewers and editors involved in this special themed issue, the *Frontiers in Psychiatry* editorial staff, and the funding institutes/programs for their contribution to advance the science and translational aspects of placebo research.

## Author contributions

PF and CS wrote the initial draft. JN and LC provided critical revisions to the manuscript. All authors approved the submitted version.

## Funding

This work was supported by the National Institute on Alcohol Abuse and Alcoholism (NIAAA) grant 1R13AA028424-01, the transregional DFG (German Research Foundation) collaborative research Treatment Expectation TRR 289 grant, and Samueli Foundation, and Society for Interdisciplinary Placebo Studies (SIPS).

## Conflict of interest

The authors declare that the research was conducted in the absence of any commercial or financial relationships that could be construed as a potential conflict of interest.

## Publisher's note

All claims expressed in this article are solely those of the authors and do not necessarily represent those of their affiliated organizations, or those of the publisher, the editors and the reviewers. Any product that may be evaluated in this article, or claim that may be made by its manufacturer, is not guaranteed or endorsed by the publisher.
